# Initial Experience of ^18^ F-FET PET-MR Image Fusion for Evaluation of Recurrent Primary Brain Tumors

**DOI:** 10.1055/s-0043-1771282

**Published:** 2023-09-06

**Authors:** Habibollah Dadgar, Manouchehr Seyedi Vafaee, Amirreza Khorasanchi, Parastoo Kordestani Moghadam, Reza Nemati, Hossein Shooli, Esmail Jafari, Majid Assadi

**Affiliations:** 1 Cancer Research Center, RAZAVI Hospital, Imam Reza International University, Mashhad, Iran; 2 Department of Nuclear Medicine, Odense University Hospital, Odense, Denmark; 3 Translational Neuroscience, BRIDGE, University of Southern Denmark, Odense, Denmark; 4 Department of Psychiatry, Odense University Hospital, Odense, Denmark; 5 Social Determinants of Health Research Center (Division of Cognitive Neuroscience), Lorestan University of Medical Sciences, Khorramabad, Iran; 6 Department of Neurology, Bushehr Medical University Hospital, School of Medicine, Bushehr University of Medical Sciences, Bushehr, Iran; 7 The Persian Gulf Nuclear Medicine Research Center, Department of Molecular Imaging and Radionuclide Therapy (MIRT), Bushehr Medical University Hospital, School of Medicine, Bushehr University of Medical Sciences, Bushehr, Iran

**Keywords:** magnetic resonance imaging, positron emission tomography, amino acid PET, brain tumor, ^18^ F-FET PET/CT

## Abstract

**Background** An accurate monitoring technique is crucial in brain tumors to choose the best treatment approach after surgery and/or chemoradiation. Radiological assessment of brain tumors is widely based on the magnetic resonance imaging (MRI) modality in this regard; however, MRI criteria are unable to precisely differentiate tumoral tissue from treatment-related changes. This study was conducted to evaluate whether fused MRI and O-(2- ^18^ F-fluoroethyl)-L-tyrosine ( ^18^ F-FET) positron emission tomography (PET) can improve the diagnostic accuracy of the practitioners to discriminate treatment-related changes from true recurrence of brain tumor.

**Methods** We retrospectively analyzed ^18^ F-FET PET/computed tomography (CT) of 11 patients with histopathologically proven brain tumors that were suspicious for recurrence changes after 3 to 4 months of surgery. All the patients underwent MRI and ^18^ F-FET PET/CT. As a third assessment, fused ^18^ F-FET PET/MRI was also acquired. Finally, the diagnostic accuracy of the applied modalities was compared.

**Results** Eleven patients aged 27 to 73 years with a mean age of 47 ± 13 years were enrolled. According to the results, 9/11 cases (82%) showed positive MRI and 6 cases (55%) showed positive PET/CT and PET/MRI. Tumoral recurrence was observed in six patients (55%) in the follow-up period. Based on the follow-up results, accuracy, sensitivity, specificity, positive predictive value (PPV), and negative predictive value (NPV) were 64, 85, 25, 67, and 50%, respectively, for MRI alone and 91, 85, 100, 100, and 80%, respectively, for both PET/CT and PET/MRI.

**Conclusion** This study found that ^18^ F-FET PET-MR image fusion in the management of brain tumors might improve recurrence detection; however, further well-designed studies are needed to verify these preliminary data.

## Background

Primary brain tumors are diagnosed by neurological examination and neuroimaging using conventional modalities; however, they are often complex and sophisticated for precise interpretation by physicians. MRI is the gold standard modality and has a high spatial resolution for the diagnosis of brain tumors 1 2 ; however, it cannot differentiate tumoral tissue from non-neoplastic changes such as edema, postoperative changes, and radiation necrosis. 3

The development of positron emission tomography (PET) radiotracers has helped overcome some of the limitations of structural MRI by improving the delineation of tumor boundaries for surgery and radiotherapy planning, differentiating between tumor progression and treatment-related responses, and monitoring tumor response to therapy. ^18^ F-fluorodeoxyglucose ( ^18^ F-FDG) PET/CT was previously suggested for the detection of brain tumors based on their elevated glucose metabolic rate. It is difficult to differentiate the brain tumor from healthy tissue using ^18^ F-FDG PET due to the high background uptake of glucose by normal brain tissue. Therefore, imaging using amino acid tracers such as ^11^ C-methionine ( ^11^ C-MET), ^18^ F-fluorophenylalanine ( ^18^ F-DOPA), or ^18^ F-fluoro-ethyl-tyrosine ( ^18^ F-FET) is used to delineate the tumor lesion from the normal brain tissue with high accuracy. 4 5 6 7 8 9

Radioactively labeled amino acids such as ^18^ F-FET have attracted scientists' interest in measuring the treatment response in brain tumor. 10 In comparison with ^18^ F-FDG PET, the lower uptake of radiotracer in the normal brain cells is because of the higher accumulation of amino acid tracers in the tumor via amino acid transporters, which is observed in the tumoral cells. Moreover, FET has a lower uptake in inflammatory cells. 11

This study was conducted to assess the value of fused ^18^ F-FET PET/MR for detecting residual tumor compared to MRI alone in patients with brain tumors.

## Methods

### Study Design and Participants

In total, 11 patients with brain tumors confirmed histopathologically who had undergone ^18^ F-FET PET in our institution for investigation of tumor recurrence from August 2016 to October 2020 were retrospectively studied. The baseline characteristics of patients are presented in Table 1 . The time interval between surgery and imaging was 3 to 4 months and the interval between MRI and PET scan was 1 week. MRI was scheduled every 3 to 4 months. The patients were followed up for 1 year or until death. The reference of recurrence was based on all available data in terms of the results of follow-up scans, later histopathological findings, and relevant clinical information; thus, the final impression was based on our expert neuro-oncology team consensus. Informed consent was obtained from all participants. All reported evaluations were performed based on the principles of the Helsinki Declaration and according to the national regulations. Retrospective analysis was performed according to ethical guidelines of the ethics commission of our university.

**Table 1:** Overview of the included patients with brain tumor

Patient	Age (y)	Sex	Therapy before PET/CT	Diagnosis based on histology
1	40	M	None	Anaplastic oligodendroglioma (WHO grade III)
2	51	M	CH and RT	Glioblastoma multiform
3	73	M	None	Low-grade oligodendroglioma (WHO grade II)
4	38	M	RT	Low-grade glioma oligodendroglioma (WHO grade II)
5	35	M	CH and RT	Anaplastic oligodendroglioma (WHO grade III)
6	46	M	None	Primitive neuroectodermal tumor (PNET)
7	27	M	CH and RT	Oligodendroglioma (WHO grade III)
8	66	F	None	Glioblastoma multiform
9	50	M	OP	Anaplastic astrocytoma (grade III)
10	56	M	CH and RT	Astrocytoma (grade III)
11	42	M	CH and RT	Glioblastoma multiform

Abbreviations: CH, chemotherapy; CT, computed tomography; OP, operation; PET, positron emission tomography; RT, radiation therapy; WHO, World Health Organization.

## Image Acquisition

### Magnetic Resonance Imaging Acquisition

MRI was performed using a 1.5-T scanner (Sonata, Siemens-Erlangen, Germany). T2-weighted and fast-spin echo sequences (660 milliseconds/100–110 milliseconds, 2-mm slice thickness, 3,206,240 matrices), fluid-attenuated inversion recovery (FLAIR) sequence, and T1-weighted gradient-echo sequences (repetition time [TR] = 1,860 milliseconds, echo time [TE] = 4.38 milliseconds with 1.2-mm slice thickness, 2,566,192 matrices) before and after administration of gadolinium were captured. Two expert radiologists reported the MRI data according to the response assessment in neuro-oncology (RANO) criteria in a consensus reading for potential recurrence. 12

### FET PET-CT

For FET-PET, two protocols were performed as follows: (1) 20-minute static imaging after intravenous (IV) injection of 7.3 mCi (200–250 MBq) ^18^ F-FET; (2) 40-minute dynamic brain scan postinjection (p.i.) using a PET/CT (Biograph) scanner (Siemens Medical Systems, Erlangen, Germany) according to a standardized acquisition protocol. Semi-quantitative PET data including the maximal standardized uptake value (SUV) of the tumor were compared to the background activity in the contralateral hemisphere. Iterative reconstruction was performed using the ordered subset expectation maximization (OSEM) algorithm with 128 × 128 matrices, 4.75-mm pixel size, 2-mm slice thickness, and 6 iterations with 21 subsets. Finally, registered PET/MR was carried out and reported separately. The integrated FET PET/MRI data were analyzed by two nuclear medicine specialists. All image interpreters were blinded to the patients' data. 13

### Statistical Analysis

All of the statistical analyses were carried out using the SPSS software version 21. Continuous data are presented as mean and standard deviation (SD). Descriptive and frequency data were analyzed using the chi-square test. A *p* -value of less than 0.05 was considered significant. Accuracy, sensitivity, specificity, positive predictive value (PPV), and negative predictive value (NPV) were calculated according to the follow-up results. The final judgment of recurrence was based on histopathology when accessible and on follow-up clinical and/or imaging data for the patients where it was not available. Tumor progression clinically or on imaging was designed as recurrence.

## Results

Eleven patients aged 27 to 73 years with a mean age of 47 ± 13 years and a positive history of craniotomy were referred to us for imaging to investigate recurrence after 3 to 4 months of tumor surgery. Detailed characteristics of patients are presented in Table 1 .

According to the results, 9/11 cases (82%) showed positive MRI and 6 cases (55%) showed positive PET/CT and PET/MRI.

**Fig. 1:**
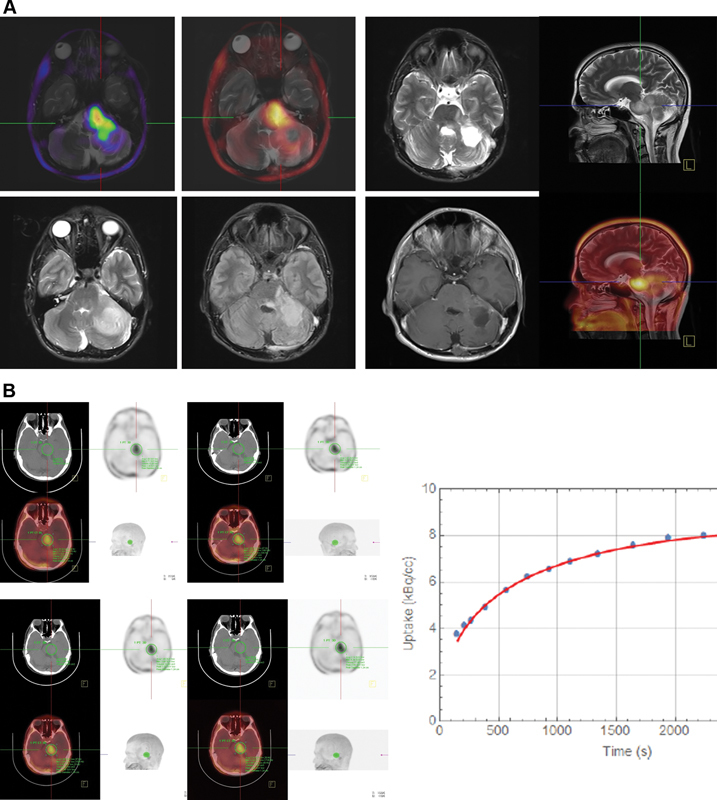
A 56-year-old man with astrocytoma (grade III) that was suspicious for recurrence. ( **A** ) There was a hyperactive lesion on the left side of the pons and the left cerebellar hemisphere. Moreover, there was an extensive hypoactive zone in the most cephalic part. The lesion was 63 × 27 mm ^2^ with a SUVmax of 4.96 (contralateral side 1.39). Magnetic resonance (MR) and positron emission tomography (PET) images were merged. The study showed recurrence or residual disease on the left side of the pons and the left cerebellar hemisphere. ( **B** ) Time-activity curves (TAC) were obtained from the dynamic images at 5 to 40 minutes, which increased until the end of the acquisition indicating a low-grade astrocytoma.

**Fig. 2:**
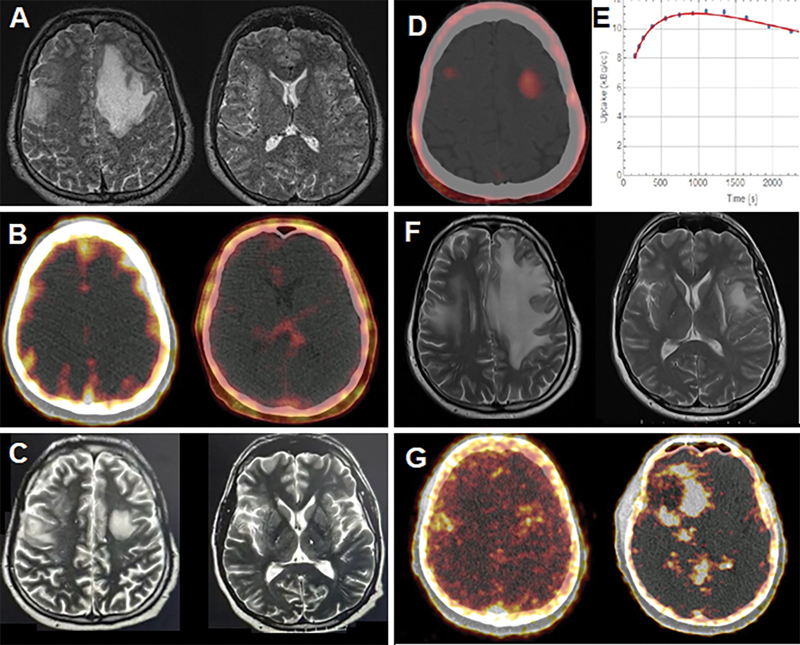
A 42-year-old patient with a high-grade glioma tumor in the left frontal lobe. ( **A** ) The patient underwent surgery in combination with chemoradiation immediately. ( **B** ) Follow-up magnetic resonance imaging (MRI) and ^68^ Ga-DOTATATE positron emission tomography (PET)/computed tomography (CT) within 1 month postsurgery were normal. Three months later, while the patient was clinically stable and ( **C** ) his scheduled MRI was unremarkable, ( **D** , **E** ) monitoring static and dynamic ^18^ F-FET PET showed multiple uptake foci in addition to the tumor bed. Time-activity curve (TAC) done over 5 to 40 minutes as dynamic imaging was compatible with high-grade glioma. The patient was under observation for up to 4 months. ( **F** ) An MRI was performed 4 months later due to worsening of the patient's condition, which showed recurrence in the left frontal lobe. Debulking surgery was performed for the second time. A second ^68^ Ga-DOTATATE PET/CT study was also done, which revealed some foci of somatostatin receptors (SSTR) uptake. ( **G** ) The patient was scheduled for peptide receptor radionuclide therapy (PRRT).

In the follow-up of the patients who were enrolled in the present study, four patients who had positive FET PET results underwent chemotherapy with temozolomide or Avastin for 60 days ( Fig. 1 ). Two patients who had positive ^18^ F-FET PET/CT findings underwent reoperation 3 months after PET acquisition, which showed local and distant metastases ( Fig. 2 ).

Two cases had at least one more type of PET/CT; case number 6 underwent FDG and ^68^ Ga-DOTATATE and case number 11 underwent ^68^ Ga-DOTATATE PET imaging. Peptide receptor radionuclide therapy (PRRT) is performed in our dedicated theranostics center for the treatment of brain tumors and the candidates should be checked for somatostatin receptors (SSTR) using ^68^ Ga-DOTATATE before inclusion ( Fig. 2 ). Tumor recurrence occurred in six patients (55%) according to the follow-up.

Based on the follow-up results, accuracy, sensitivity, specificity, PPV, and NPV were 64, 85, 25, 67, and 50%, respectively, for MRI alone and 91, 85, 100, 100, and 80%, respectively, for both PET/CT and PET/MRI ( Table 2 ).

**Table 2:** The results of accuracy, sensitivity, specificity, positive predictive value (PPV) and negative predictive value (NPV) according at follow-up

	Accuracy	Sensitivity	Specificity	PPV (%)	NPV (%)
MRI	64	85	25	67	50
PET/CT	91	85	100	100	80
PET/MRI	91	85	100	100	80

Abbreviations: CT, computed tomography; MRI, magnetic resonance imaging; PET, positron emission tomography.

The plot of the SUVmax from the regions of interest (ROIs) of ^18^ F-FET images in recurrent high-grade revealed an early peak of radiotracer within 5 and 15 minutes p.i. followed by a reduction ( Fig. 2 ), but a slower uptake peak toward the end of the scan has been shown in low-grade tumors ( Fig. 1 ). The dynamic PET results of two positive scans are presented in Figs. 1 and 2 . Case number 10 had a primary grade III astrocytoma, but dynamic imaging was compatible with a low-grade tumor, which may be due to the nonhomogeneous context of the tumor ( Fig. 1 ). However, case number 11 showed a high-grade glioma (HGG), which was similar to its primary tumor ( Fig. 2 ). Uptake changes over time up to the first 20 minutes for low- and high-grade tumors were similar in glioblastoma and astrocytoma cases; however, absolute values were higher in the former.

## Discussion

The necessity of combining molecular/metabolic imaging data into the diagnosis and treatment of patients with brain tumors is more and more appreciated. There is still no available imaging modality for unequivocal differentiation between treatment-induced changes and tumor recurrence in the brain, even with advanced MRI techniques. 14

In the field of neuro-oncology, conventional gadolinium-enhanced MRI is the standard imaging technique for diagnostic assessment of brain tumors. 15 However, standard MRI sequences are not sufficient for differential diagnosis, tumor grading, tumor delineation, treatment response evaluation, and discrimination between therapy-associated changes and tumor tissue. 16 Another main drawback of MRI is its failure to discriminate a tumor from an edema or a gliosis in the nonenhancing parts of brain tumors. 17 This is a considerable challenge because almost 40 to 45% of nonenhancing primary brain tumors turn out to be malignant, and full characterization of different tumor areas is pivotal for managing of cancerous patients. 17 Due to such gadolinium enhancement drawbacks especially for the distinction between treatment-induced changes and tumour recurrence, some advanced functional imaging techniques, that is, perfusion MRI, dynamic susceptibility contrast, dynamic contrast-enhanced, arterial spin labeling, diffusion-weighted imaging, MR spectroscopy, and chemical exchange saturation transfer have been implemented for a better assessment of brain tumors. However, newer advanced and innovative MRI techniques are not widely available. Such issues justify the necessity for molecular imaging using PET tracers in the clinical management of brain tumors, able to afford additional insights into tumour pathophysiology. 18

PET/MR may supply added diagnostic value and apparently reaches the maximum precision if combined with advanced MR procedures, augmenting the physician's confidence. Nevertheless, notwithstanding the evident benefits, the distribution of the PET/MR technique is still very limited possibly due to the economic costs. The cost of the PET/MRI system is roughly 3 to 4 times higher than that of a PET/CT scan. 19

In comparison of PET/MRI and PET/CT, one of the main advantages of PET/MRI over PET/CT is the deletion of the ionizing radiation of the CT component, which is more important in the pediatric groups than in adult patients, especially when multiple follow-up scans are required.

In addition, MRI has a much superior soft-tissue resolution, which permits more detailed imaging compared to the CT scan. It vividly permits better diagnostic images of the tumor structures. 19

Combined PET/MRI acquainting data concurrently in space and time as compared with sequential PET or MRI acquisitions, which are done in this study, permits shorter scanning time, which lessens the patient's stress and the staff's workload and diminishes the occurrence of image misregistration. 19

In this regard, FET PET has been shown to be a powerful tool for differentiating postoperative lesions from tumor recurrence and for grading brain tumors. 13 20 21 The results of the present study are in agreement with several previous studies using ^18^ F-FET for tumor recurrence evaluation. 22 23 The increased diagnostic accuracy may suggest that the application of the fused PET/MRI might be favorable for decision-making in routine practice compared to the sole use of MRI examination. The equivocal description of T2 progress frequently leaves practitioners with uncertainty. The present study indicates that fused FET PET/MRI might be especially useful in these equivocal cases.

In this respect, Dunet et al 24 in a systematic review compared ^18^ F-FET and ^18^ F-FDG PET for diagnosis of brain tumors. They showed sensitivity and specificity of 94 and 88% for ^18^ F-FET and 38 and 86% for ^18^ F-FDG, indicating the superiority of ^18^ F-FET in diagnosis of brain tumor. Although both ^18^ F-FET and ^11^ C-MET showed similar uptake and diagnosis in glioma and metastases, the short half-life of ^11^ C (20 minutes) is a major limitation of clinical use of ^11^ C-MET in neuroimaging. 25

MRI does indeed have difficulty in detecting the extent of brain tumor. This is due to the fact that the tumor borders often extend beyond the contrast enhancement of Gd-DTPA and the tumor cannot be distinguished from the edema in this area. 1 2 25 Also, the tumor regions necrosed by irradiation have a negative impact on the results of ^18^ F-FET PET. 26 Therefore, according to the clinical evidence, a combination of MRI and ^18^ F-FET PET could improve the interpretation of images. Some studies found a higher sensitivity for the postoperative residual brain tumor using ^18^ F-FET PET compared to MRI. 8 11 27 28

Functional imaging modalities like amino acid ligand integrated PET/MR may be useful for delineation of the tumor margins following surgery. In the present study, the fused PET/MR system provided more accurate information compared to MRI in four patients, indicating the influential role of this modality in the management of this challenging field of neuro-oncology.

Previous studies found the higher diagnostic value of fused MRI and PET over any single imaging technique. 29 Therefore, a fused PET/MRI system might have an extra benefit because of the co-interpretation of both signals. 29

The findings of the present study were consistent with previous investigations. A meta-analysis found that ^18^ F-FET PET or PET/CT had pooled sensitivity and specificity values of 82 and 80% in discrimination between brain tumor recurrence and radiation necrosis after therapy, respectively. In the subgroup of subjects with doubtful glioma recurrence, the sensitivity and specificity values were 83 and 81%, respectively. 30 Similar findings were also reported by Furuse et al who found higher diagnostic values of ^18^ F-FET PET or PET/CT compared to ^18^ F-FDG and ^11^ C-methionine PET or PET/CT. 31 Kim and Ryul Shim found that amino acid PET or PET/CT, including ^18^ F-FET PET, had good statistical parameters in discriminating between residual or recurrent brain tumor and treatment-related changes (pseudoprogression) in the cases with HGGs. 32

In short, the present study showed that FET-PET could be potentially used to differentiate low-grade brain tumors from high-grade ones, which is in agreement with previous studies. 33 34 However, tumor grading using ^18^ F-FET PET is still controversial. 11 27 28 35 36

A meta-analysis found that ^18^ F-FET PET or PET/CT had higher sensitivity but lower specificity compared to ^18^ F-FDG PET or PET/CT for discriminating between HGGs and LGGs; however, the diagnostic values were similar to those of ^11^ C-methionine PET or PET/CT in this regard. 37

According to our results, in low-grade tumors, ^18^ F-FET uptake increases from the time of radiotracer injection to the end of the acquisition, whereas in patients with high-grade tumors, uptake peaks 5 to 15 minutes after injection and then decreases until the end of the acquisition. The behavioral differences in the kinetics of high- and low-grade tumors are also affected by several factors. Researchers 8 suggest that the higher early uptake of radiotracer in high-grade tumors is because of the higher density of intratumoral vessels and higher angiogenesis leading to more local blood volume in patients with progressive malignancy. 22 A study 23 found that upregulation of facilitated amino acid transport, which is responsible for increasing in accumulation of ^18^ F-FET in gliomas, occurs with an increase in the expression of amino acid transporter in vessels of the tumor. 36 38 It has been recently reported that close relation between enhanced uptake of amino acid and angiogenesis in gliomas may lead to higher early radiotracer accumulation in high-grade tumors than in low-grade tumors. 13 It still is not clear how ^18^ F-FET is retained within the cells. 25 Therefore, HGGs show stable or a slight reduction in the uptake of radiotracer and LGGs show a slight enhancement in the uptake until the end of imaging. Moreover, it has been shown that the interruption of the blood–brain barrier in HGGs may facilitate the passive back diffusion of radiotracer and may be the reason for the earlier uptake reduction in high-grade tumors than in low-grade tumors. 8

Qualitative and semiquantitative uptake parameters of ^18^ F-FET significantly influence the clinical outcome. Moreover, time-activity curves in the kinetic analysis are highly prognostic for malignant transformation. In gliomas with positive ^18^ F-FET, reduction in the time-activity curve in the dynamic scan may be considered as an undesirable prognostic factor for astrocytic LGGs and may ease treatment decisions by identifying high-risk patients.

Moreover, using imaging as a valuable diagnostic method, for the distinction of LGGs and HGGs would improve the management of patients with brain malignancy. It should be mentioned that the demonstration of tumor burden and evaluation of treatment response are limited in World Health Organization (WHO) grade I and II gliomas and a major percentage of grade III gliomas due to their contrast nonenhancing on MRI. Therefore, using MRI for the distinction between LGGs and HGGs is not a straightforward task. In a few studies, the role of amino acid PET has been evaluated for the distinction between LGGs and HGGs according to the value of tracer accumulation. 8 25 26 ^18^ F-FET PET imaging offers a preferable distinction parameter according to the level of tracer accumulation in HGGs and LGGs. Time-activity curves with SUVmax-related time can predict the level of the tumor; for example, grade II tumors show a slow, steady enhancement versus the fast accumulation of amino acid tracers by grade III and IV tumors. Therefore, a dynamic PET in comparison to static PET scan can more accurately differentiate HGGs from LGGs. 28 35 36 38 39

This study found the behavioral difference in the ^18^ F-FET uptake kinetic between recurrent HGGs and LGGs. Although the results are promising, a larger sample size is needed for confirmation of results. Therefore, in the management of recurrent gliomas, there is a clinical requirement to introduce an efficient imaging modality to improve clinical outcomes. In this study, we showed the combination of MRI and PET as hybrid PET/MRI is an efficient technique in the assessment of recurrent gliomas providing additional data in equivocal cases according to the RANO classification.

## Limitations

The present research has some limitations. The main limitation was the small sample size, which was difficult to overcome given the scarcity of FET PET/CT, especially in a limited resource environment. Second, the study had a retrospective design and the patients were referred from different parts of the country, so their physicians followed PET acquisition. Third, follow-up was not long enough for long-term assessment of the patients' clinical outcomes, especially considering the imaging data. Fourth, we could not perform dynamic imaging to obtain time-activity curves in all positive participants. Fifth, our participants had different types of brain tumors, which could affect the results of the study. Larger multicenter prospective studies are required to confirm the findings of the present study.

## Conclusion

This study found that ^18^ F-FET PET-MR image fusion in the management of brain tumors might improve recurrence detection. It also demonstrated that imaging biomarker characteristics obtained from dynamic and semiquantitative techniques using ^18^ F-FET PET-MRI for evaluation of structural/molecular brain alterations can be considered a holistic approach to such challenges in neuro-oncology. In addition, it can be mentioned that PET/CT is superior to MRI alone. But if PET/MRI is available, in this case it is preferable due to radiation protection issues and better soft-tissue resolution of MRI compared to CT. However, further well-designed studies are needed to confirm these preliminary data.
